# Measuring optic nerve sheath diameter in children: a simple ultrasound protocol for ICP assessment

**DOI:** 10.3389/fped.2025.1741048

**Published:** 2026-01-22

**Authors:** Bogdana Sabina Zoica, Bipin Chalattil

**Affiliations:** 1 Pediatric Intensive Care Unit, King’s College Hospital, London, United Kingdom; 2 Intensive Care, Great Ormond Street Hospital for Children, London, United Kingdom

**Keywords:** ICP, ONSD, optic nerve sheath diameter, raised intracranial pressure, traumatic brain injury, point-of-care ultrasound, POCUS

## Abstract

Prompt and accurate detection of elevated intracranial pressure (ICP) is vital in the management of pediatric traumatic brain injury (TBI). While invasive ICP monitoring remains the gold standard, its application is often limited by contraindications or local logistical constraints. Consequently, a substantial number of moderate TBI cases are managed without direct ICP monitoring, despite the risk of secondary intracranial hypertension. This underscores the need for reliable, non-invasive diagnostic alternatives. One such technique—optic nerve sheath diameter (ONSD) measurement via point-of-care ultrasound (POCUS)—leverages cerebrospinal fluid (CSF) accumulation around the retrobulbar optic nerve as a surrogate marker for raised ICP. Although ONSD is recognised for its simplicity, speed, and repeatability, its clinical adoption in pediatric settings remains limited due to the absence of standardised guidelines and normative data. This manuscript synthesises the current evidence on ONSD measurement in children, highlighting its diagnostic potential, methodological considerations, and limitations. By consolidating recent research, we aim to support pediatric intensivists in the practical application of ONSD as a non-invasive tool for ICP assessment, ultimately improving clinical decision-making and prognostic evaluation in pediatric TBI.

## Introduction

Raised intracranial pressure (ICP) is closely related to clinical outcomes in pediatric patients with traumatic brain injury (TBI) and has been identified as an important prognostic factor.

Managing patients with neurocritical illness requires multimodal neuromonitoring and immediate management of elevated ICP. Invasive ICP measurements are presently the gold standard for the initial diagnosis and follow-up assessment of ICP (1). However, reliable non- invasive techniques, such as fundoscopy, tympanic membrane displacement, transcranial Doppler (TCD), optic nerve sheath diameter (ONSD), computed tomography (CT), and magnetic resonance imaging (MRI) are needed owing to contraindications for invasive ICP measurement, local issues, or as a part of a primary assessment and ongoing evaluation in an acute setting such as an emergency service or an intensive care unit (ICU) (2–5). This article focuses on the technique for measuring the optic nerve sheath diameter (ONSD) using B-mode ultrasound for the diagnosis, monitoring, and management of elevated ICP in children.

## Anatomy and physiology

**Figure 1:**
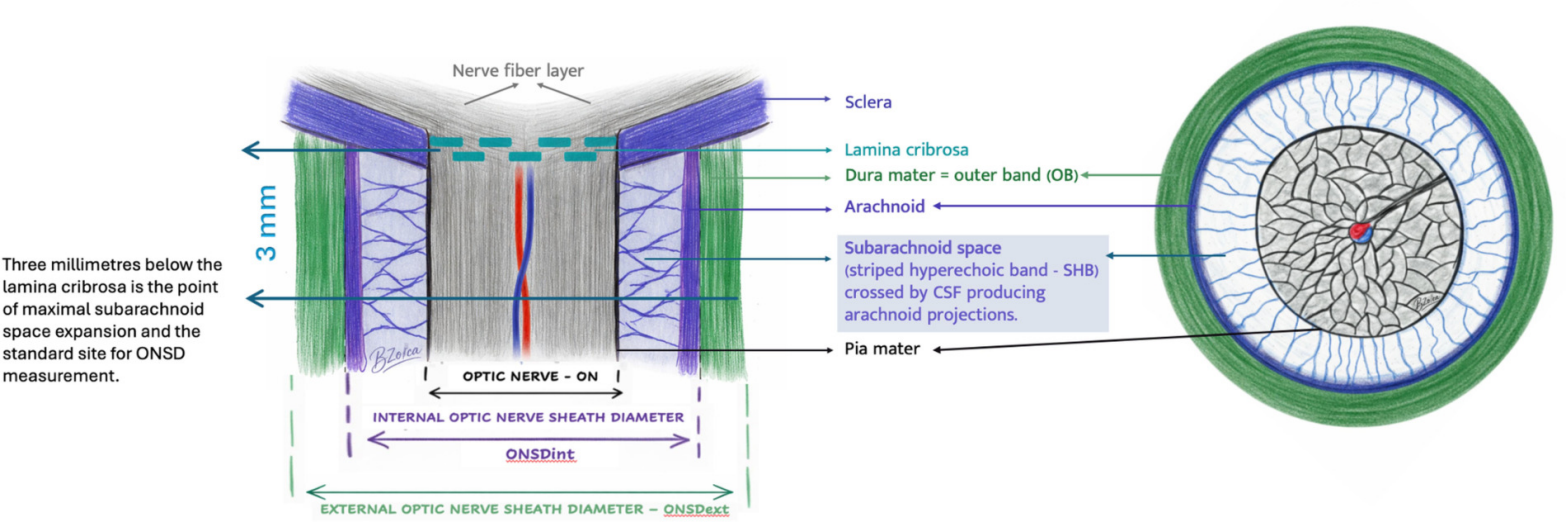
Sagittal and transverse sections of relevant optic nerve anatomy showing the standardised ultrasound measurement site for optic nerve sheath diameter (ONSD), located 3 mm posterior to the lamina cribrosa.

The eye provides an ideal acoustic window for point-of-care ultrasound (POCUS) due to its superficial location and well-defined, fluid-filled compartments, which facilitate high-resolution imaging. Enclosed within the Tenon's capsule—a thin, membranous sac—the globe is cushioned by periocular fat and soft tissues and is protected by the bony orbit. Anatomical connectivity is maintained via the corneoscleral junction anteriorly and the optic nerve (ON) posteriorly, which is a critical structure for assessing ICP (Figure 1).

Surrounding the posterior globe, the extraocular muscle tendons traverse the Tenon's capsule to insert into the sclera, enabling multidirectional ocular movement. Within the globe, the anterior chamber (AC) and posterior chamber (PC) are two key anechoic, fluid-filled structures that must be identified during sonographic evaluation. The crystalline lens separates these chambers and serves as a crucial landmark in determining the optimal scanning planes. Posterior to the PC lies the retina, encased within a hyperechoic neural membrane, which further delineates the posterior ocular anatomy (6).

A critical anatomical landmark when performing ONSD measurements is the lamina cribrosa (LC)— a perforated region of the sclera through which the ON exits the globe. This dense, fibrous structure produces a strong hyperechoic signal and can create posterior acoustic shadowing on ultrasound, potentially obscuring or falsely elongating the sheath margins. It is essential to recognise this artefact to avoid misidentification of the anterior margin of the ON (7).

Understanding these structures is crucial when performing ONSD measurements via ultrasound, particularly in pediatric patients, where variations in anatomical proportions can affect image interpretation. This anatomical knowledge ensures accurate localisation of the ON entry and sheath, facilitating reliable and reproducible ONSD measurements (6).

The ON is visualised on ultrasound as a structure lying posterior to the retina and optic disc, with a hypoechoic central core and hyperechoic margins representing the optic nerve sheath (ONS) (Figure 1). The sheath is an extension of the dura, arachnoid, and pia mater, fully enclosing the intraorbital portion of the ON (8–10). The pia mater cannot be resolved sonographically but the subarachnoid space is trabeculated, hyperechoic in comparison with the optic nerve and often has a honey comb pattern. This space is continuous with the intracranial compartment, allowing cerebrospinal fluid (CSF) to reflect ICP changes along the sheath (11). This anatomical relationship enables the ONS to distend in response to increased ICP dynamically. Both *in vivo* and cadaveric studies with artificially induced ICP elevations have shown that the most responsive and distensible portion of the sheath lies approximately 3 mm behind the posterior globe. Consequently, this location is now the standard site for measuring ONSD (6, 11). The variability of this distance in infants and young children has not been systematically studied; however, it is plausible that the optimal measurement point lies closer to the globe in smaller eyes.

At this level, the subarachnoid space (striped hyperechoic band - SHB) is bounded externally by the dura (outer band – OB) and retroorbital fat. Internal ONSD (ONSDint) only includes SHB in the measurement, whereas external ONSD (ONSDext) includes both SHB and OB. ONSDint and ONSDext have similar accuracy in detecting elevated ICP. Performing both ONSDint and ONSDext on all measurements did not have enough support, as it is not pragmatic. However, the two measurements differ by up to 1.5 mm— a clinically and methodologically significant discrepancy. Researchers must therefore state explicitly which measurement was used and, where feasible, report both. (2) Precise reporting is essential to generate robust, comparable datasets that can be reliably pooled and interpreted in reviews and meta-analyses.

ONSD shows a linear increase in correlation with rising ICP, up to around 7.5 mm, beyond which further expansion plateaus (12). However, there is significant age-dependent variability in ONSD values among children, making it difficult to establish a universal cutoff for raised ICP in pediatric populations. Ongoing research continues to explore age- and size-adjusted reference values to enhance clinical interpretation in this group (13–17).

## Indications and contraindications

ONSD assessment is currently regarded as the most effective non-invasive method for dynamically estimating ICP, showing strong correlation with invasive ICP measurements (18–20). An elevated ONSD reliably indicates increased ICP, with studies demonstrating a sensitivity of approximately 95.6% and specificity of 92.3% at a cutoff value of 5 mm (21). A commonly cited formula to estimate non-invasive ICP (nICP) based on ONSD is: nICP- ONSD = 5.00 × ONSD - 13.92 mmHg. However, this formula was derived from adult populations and has not yet been validated for use in pediatric patients (22). Therefore, it should be applied cautiously in children, taking into account their anatomical and physiological differences.

ONSD ultrasound has been found helpful in a wide range of clinical scenarios, including the evaluation of idiopathic intracranial hypertension, intracranial hypotension, hypoxic-ischemic encephalopathy, stroke, intracranial hematoma, traumatic brain injury, shunt evaluation, and in perioperative neurosurgical monitoring (22). It is considered a safe and repeatable technique with minimal risk. Nonetheless, when an open globe injury is suspected, ONSD measurement should not be performed without consultation from an ophthalmologist, as inadvertent pressure on the eye could lead to further ocular damage (23).

## Materials and equipment

Commercially available high-frequency ocular ultrasound units are designed specifically for dedicated studies of the eye; however, most standard ultrasound machines equipped with high-frequency linear array probes (typically ≥7.5 MHz) are sufficient to visualise the ON using the closed-eye technique (24, 25). Additional equipment may include a clear, sterile intravenous catheter dressing to act as a barrier, along with ultrasound gel as a coupling medium. A trained individual can perform the examination and generally does not require additional assistance for patient positioning.

## Methods

### Procedure

The acquisition and measurement approach described below aligns with the quality standards proposed by Hirzallah et al., including probe selection, safety parameters, patient positioning, standardized imaging planes, and measurement site (2).

1. Patient Preparation (Awake vs. Sedated):
  (a) In awake and cooperative children, briefly explain the procedure to reduce anxiety.
  (b) In obtunded, intubated, or deeply sedated patients, perform a gross eye examination to rule out globe rupture or hyphema. If signs of globe rupture are present (e.g., visible conjunctival or scleral defect, 360-degree conjunctival haemorrhage, severe chemosis, hypotony, or total hyphema), abort the procedure and consult an ophthalmologist (23).

Positioning and Eye Orientation
  (a) Position the child supine with the head elevated 20–30 degrees (“head-up” position), as head angle affects both ICP and ONSD (2).
  (b) Instruct awake children to “look straight ahead” with their eyes closed.
  (c) In sedated patients, assess baseline eye orientation to ensure the ultrasound beam aligns in the transverse plane and avoids oblique sections of the ON.

**Figure 2:**
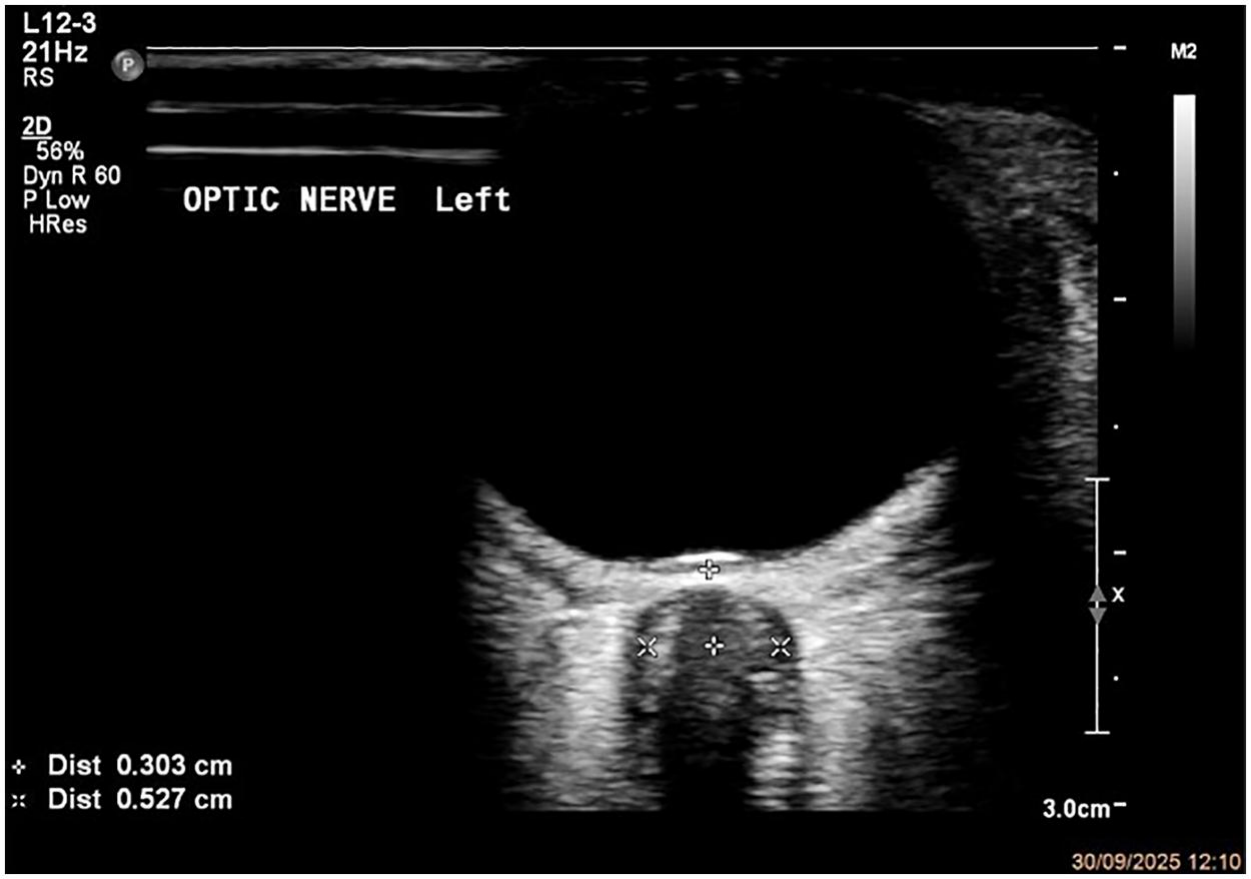
Measurement of optic nerve sheath diameter (ONSD). Transverse B-mode image of the left ON, obtained 3 mm posterior to the globe. Callipers measure the ONSDint between the inner margins of the hyperechoic ONS.

2. Preparation of the Ocular Surface:
  (a) There are differences in the practice of using a transparent barrier. In Hirzallah et al., 69.7% of studies did not report whether adhesive dressings were used, and 18.2% explicitly stated that they did not use them (25).
  (b) If using a transparent barrier (e.g., sterile adhesive dressing), you may apply a thin layer of petroleum jelly or ophthalmic ointment to the closed eyelid to prevent air entrapment beneath the barrier, which can degrade image quality. Then, apply ultrasound gel on top of the barrier to serve as the acoustic coupling medium.
  (c) If no barrier is used, skip the ointment and apply ultrasound gel directly to the closed eyelid surface.
3. Ultrasound settings and probe selection
  (a) Use a high-frequency linear array probe (≥7.5 MHz) in B-mode.
  (b) Apply the ALARA principle to minimise energy exposure, aiming for a mechanical index (MI) <0.23, thermal index (TI) ≤1, and acoustic output ≤ 50 mW/cm^2^ (4, 24).
  (c) Standardise gain, brightness, and contrast across examinations.
4. Image acquisition
  (a) Rest your scanning hand on a stable surface such as the patient's nose, midface, or forehead.
  (b) Gently place the probe on the closed upper eyelid in the transverse (axial) orientation.
  (c) Use small probe movements in nasal-temporal and cephalad-caudad directions to align the ON with the visual axis, including the AC, PC, lens, and retina. Adjust the gain to differentiate the hyperechoic ONS surrounding the hypoechoic ON clearly.
  (d) When possible, the imaging axis should be in line with the ON axis, where the ON is perpendicular to the globe and demonstrate the maximum length along the image frame of the ON. Use Doppler to identify the ON through the course of the central retinal artery and vein (2).
  (e) Obtain images in the transverse plane. Sagittal plane imaging may supplement but is not required (2, 26). In obtunded patients where eye position is uncertain, if clinically feasible, examine the contralateral eye to estimate pupil orientation on the studied eye (27).
5. ONSD measurement technique
  (a) Capturing multiple images and measurements for accuracy is advised but not mandatory (25, 28). Rescan if significant discrepancies are observed. As long as clinically feasible, always scan bilaterally.
  (b) If the LC is clearly visible, it should be used as the measuring starting point. If the LC is not visible, use the optic disc (papilla) at the retinal surface.
  (c) From the chosen reference, measure 3 mm posteriorly along the axis of the ON. This corresponds to the segment most responsive to changes in ICP (25).
  (d) Use electronic callipers to measure the ONSD by identifying the hyperechoic margins that surround the ON. Position the callipers perpendicular (at a 90-degree angle) to the long axis of the ON, exactly 3 mm posterior to the globe. This orthogonal placement is essential to avoid measurement errors and is considered a key quality standard for reliable and reproducible ONSD assessment (2, 25) (Figure 2).
  (e) In clinical use, the interface between the hyperechogenic band and hypoechoic OB should be used as the measurement reference point. This is referred to as ONSDint.
  (f) As mentioned in the anatomy section, for research purposes, investigators should consider reporting both ONSDint and ONSDext.
  (g) ONSDext utilises the OB as the measurement reference point (2, 25).
  (h) Calculating the mean ONSD from multiple measurements can be considered but is not mandatory (25, 28).
6. Quality, governance and data collection
  (a) Capture multiple images in each plane for both eyes.
  (b) If discrepancies are noted between images, repeat scanning and measurement.
  (c) Consider calculating the mean ONSD per eye, although this is not mandatory (25, 28).
  (d) Compare the measurements with established age-stratified ONSD cutoff values (Table 1), keeping in mind the variability among published studies (16, 17).
  (e) Interpret findings within the broader clinical context. Serial measurements over time may be more informative than a single isolated value.

**Table 1:** Age-Stratified pediatric ONSD cutoff values for raised ICP.

Age group (years)	ICP threshold (MM HG)	ONSD cutoff (mm)	Sensitivity (%)	Specificity (%)	Reference
≤1	≥20	4.99	50	58.8	Kerscher SR et al. (16)
>1	≥20	5.75	91.7	66.7	Kerscher SR et al. (16)
≤14	≥20	5.5	93.2 (84.9–97.8)	74 (64.3–82.3)	Padayachy LC et al. (17)

### Image quality assessment

The image chosen for ONSD measurement should demonstrate the clearest anatomical definition of the ON and its surrounding sheath. The optimal image is one where the interface between the ON head and surrounding structures is thinnest and most sharply defined. There is currently no consensus on whether inclusion or exclusion of the lens within the image field affects measurement accuracy, and its impact remains unclear (2, 25). Maintaining consistent equipment settings—such as gain, depth, and focal zones—is essential for measurement reliability and reproducibility.

**Figure 3:**
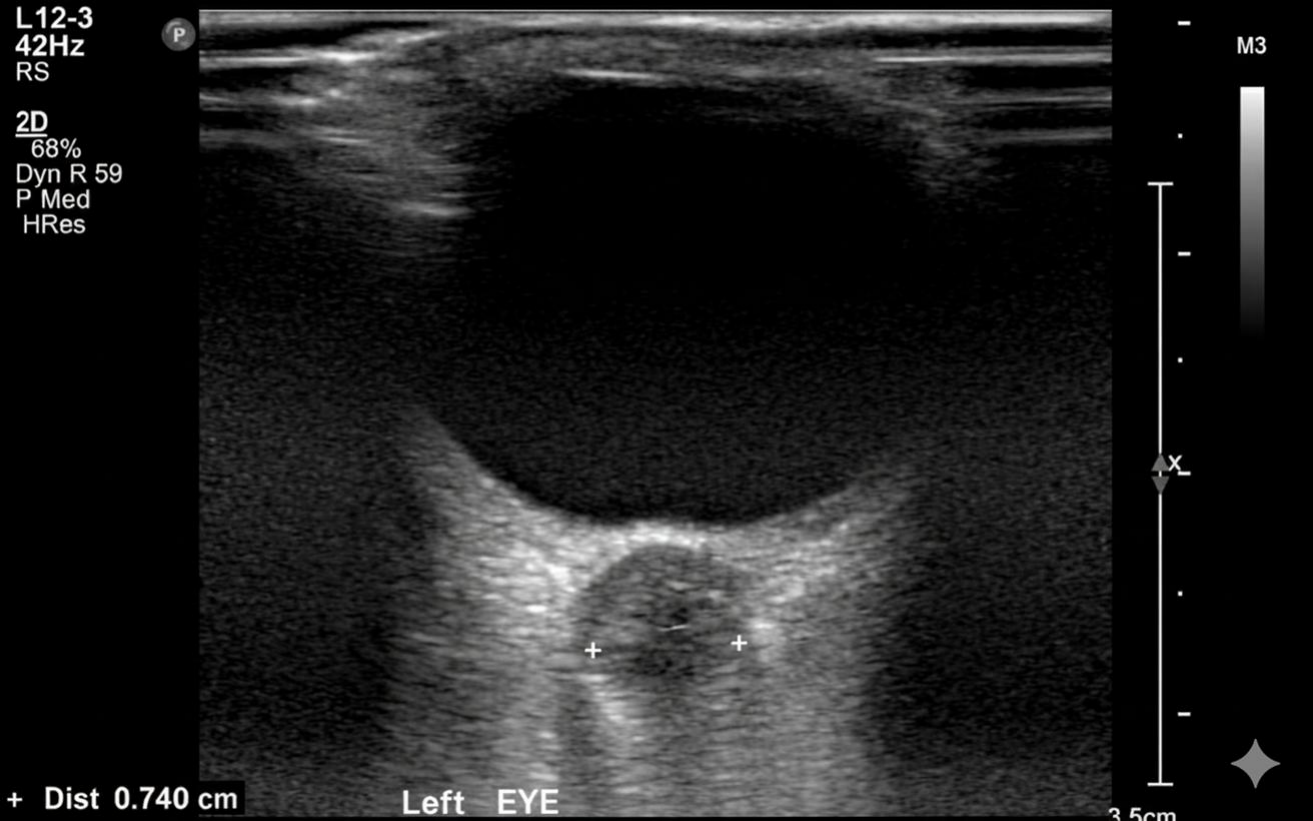
Example of blooming artefact leading to ONSD overestimation. This transverse B-mode ultrasound image of the left eye shows measurement of the ONSD at 3 mm posterior to the globe. The reported ONSD is 0.70 cm (7.0 mm). However, the hyperechoic margins of the ONS appear markedly thickened and poorly demarcated from surrounding tissue, suggesting the presence of a blooming artefact. The sheath seems disproportionately large compared to the ON itself.

Common ultrasound artefacts should be recognised and accounted for during image acquisition and interpretation. One such artefact is overgain, sometimes loosely referred to as a “blooming” effect, where excessive gain exaggerates the echogenic margins of the ONS, potentially leading to overestimation of its diameter (24) (Figure 3).

**Figure 4:**
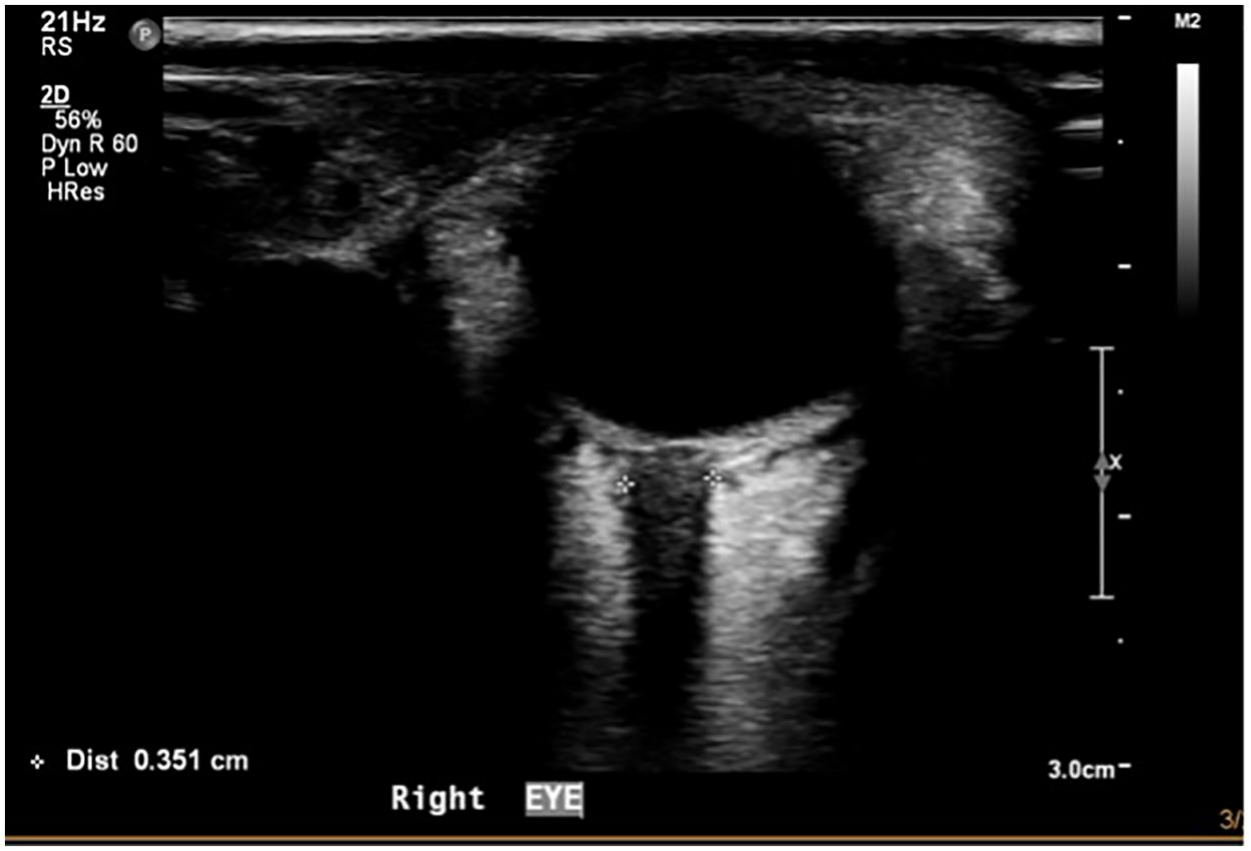
Posterior acoustic shadowing from the LC obscuring ONS margins. This transverse B-mode ultrasound image of the ON demonstrates a common artefact caused by the LC. The resulting posterior acoustic shadow creates a dark vertical band that attenuates the ultrasound beam, obscuring the lateral margins of the ONS. As a result, the external sheath boundaries are not clearly visualised, and accurate ONSD measurement cannot be reliably performed in this image. Recognising this artefact is essential to avoid misinterpretation and potential under- or overestimation of ICP.

Another common issue is acoustic shadowing. This occurs when the ultrasound beam encounters a strongly curved or dense structure, such as the LC or the posterior globe wall. The beam is refracted or attenuated, resulting in a hypoechoic or anechoic area behind the structure. This shadowing can obscure the margins of the ONS, leading to inaccurate measurements (24, 29) (Figure 4).

Proper technique, artefact recognition, and careful image selection are essential to ensure accurate and reproducible ONSD measurements.

## Results & discussion

### Limitations

ONSD is an operator-dependent measurement (24). Several authors have questioned its reliability, suggesting that some structures interpreted as the ONS in prior studies may instead represent imaging artefacts—such as posterior acoustic shadowing from the LC—rather than true anatomical boundaries. Additionally, false-positive measurements can occur due to excessive pressure on the globe or poor technique, potentially leading to unnecessary interventions.

Several anatomical and physiological factors can impact ONSD accuracy, including eye movement, axial length, myopia, intraorbital lesions, and variations in the anatomy of the optic canal within the sphenoid bone (7). Importantly, the ONS is not a simple circular structure; it is a complex compartmentalised space formed by arachnoid trabeculae, septa, and pillars within the subarachnoid space (30). Even after the intracranial pressure normalises, the sheath may remain dilated due to impaired elasticity and incomplete retraction, contributing to persistently elevated ONSD values (31).

Some authors have proposed that A-mode ultrasound may theoretically reduce the measurement artefacts seen with B-mode imaging, such as blooming or overgain effects (24). However, B-mode remains the standard modality for POCUS and is used in the overwhelming majority of clinical studies. In a systematic review by Hirzallah et al., all 357 studies included utilised B-mode imaging (25). When performed optimally with optimised gain and technique, B-mode ultrasonography can minimise artefact-related inaccuracies.

Despite its limitations, ONSD remains a practical and rapid bedside tool for assessing elevated intracranial pressure, especially when other neuromonitoring methods are unavailable or pose a significant risk. For example, in a critically ill child with a stable intracranial haemorrhage and a known ONSD baseline, serial bedside ONSD assessments may help detect dynamic changes in ICP if the child deteriorates clinically in the ICU. In such scenarios—particularly when patient transport is unsafe or would not change management unless ICP is rising—ONSD offers a valuable screening option.

Therefore, in pediatric patients at risk for raised ICP, early and repeated ONSD measurements may provide more meaningful clinical information than a single absolute value.

## Conclusion

ONSD ultrasonography is a relatively recent, non-invasive method for estimating ICP. It is a rapid and straightforward technique that can be reliably performed bilaterally by trained clinicians. Because ONSD responds dynamically to changes in ICP, it serves as a valuable adjunct to other neuromonitoring modalities in both emergency and critical care settings.

The introduction of the ONSD Point-of-Care Ultrasound Quality Criteria Checklist (ONSD POCUS QCC) provides a standardised framework for clinical implementation, training, quality assurance, and research. Clinicians planning to integrate ONSD into practice should refer to this checklist to align with current international standards and ensure consistency in measurement technique and interpretation (2).

## Data Availability

The original contributions presented in the study are included in the article/Supplementary Material, further inquiries can be directed to the corresponding author.
